# ENDOCRINOLOGY IN THE TIME OF COVID-19: Clinical management of neuroendocrine neoplasms (NENs)This manuscript is part of a commissioned series of urgent clinical guidance documents on the management of endocrine conditions in the time of Covid-19. This clinical guidance document underwent expedited open peer review by Karel Pacak (NICHD, Bethesdad, MD, USA), Simona Grozinsky-Glasberg (Hadassah-Hebrew University Hospital, Jerusalem, Israel), and Wouter de Herder (Erasmus MC, Rotterdam, The Netherlands)

**DOI:** 10.1530/EJE-20-0424

**Published:** 2020-08-01

**Authors:** Ruth T Casey, Gerlof D Valk, Camilla Schalin-Jäntti, Ashley B Grossman, Rajesh V Thakker

**Affiliations:** 1 Wolfson Diabetes and Endocrinology Clinic, Cambridge University Hospital NHS Foundation Trust, Cambridge, UK; 2 Department of Medical Genetics, Cambridge University, Cambridge, UK; 3 Department of Endocrine Oncology, University Medical Centre Utrecht, ENETS Centre of Excellence, Utrecht, Netherlands; 4 Division of Endocrinology, Abdominal Center, University of Helsinki and Helsinki University Hospital, Helsinki, Finland; 5 Royal Free Hospital ENETs Centre of Excellence, London, UK; 6 Centre for Endocrinology, Barts and the London School of Medicine, London, UK; 7 Oxford Centre for Diabetes, Endocrinology and Metabolism, University of Oxford, Oxford, UK; 8 Academic Endocrine Unit, Radcliffe Department of Medicine, University of Oxford, Oxford, UK

## Abstract

In viral pandemics, most specifically Covid-19, many patients with neuroendocrine neoplasms (NENs), including phaeochromocytomas, paragangliomas and medullary thyroid carcinoma, may develop Covid-19 in a mild or severe form, or be concerned about the influence of viral infection relative to their anti-tumoral therapy. In general, newly presenting patients should be assessed, and patients recently receiving chemotherapy, targeted therapy or radionuclide therapy, or showing tumour growth, should be closely followed. For previously diagnosed patients, who have indolent disease, some delay in routine follow-up or treatment may not be problematic. However, patients developing acute secretory syndromes due to functional neuroendocrine neoplasms (such as of the pancreas, intestine or lung), phaeochromocytomas and paragangliomas, will require prompt treatment. Patients with life-threatening Covid-19-related symptoms should be urgently treated and long-term anti-tumoral treatments may be temporarily delayed. In patients with especially aggressive NENs, a careful judgement should be made regarding the severity of any Covid-19 illness, tumour grade, and the immunosuppressant effects of any planned chemotherapy, immunotherapy (e.g. interferon-alpha), targeted therapy or related treatment. In other cases, especially patients with completely resected NENs, or who are under surveillance for a genetic disorder, a telephone or delayed consultation may be in order, balancing the risk of a delay against that of the possible development of Covid-19.

## Introduction

- SARS CoV2 viruses (Covid-19) bind to angiotensin-converting enzyme-2 (ACE2), a cell-surface receptor, and a potential interaction between Covid-19 and the renin-angiotensin-aldosterone system has been reported (1, 2, 3, 4). However, such interactions between Covid-19 or other SARS viruses and receptors expressed on neuroendocrine neoplasms (NENs), such as somatostatin receptors (SSRs) and histamine receptors (H1 and H2 receptors), have not been reported.
- The specific effects of Covid-19 infection in patients with NENs have not as yet, to our knowledge, been reported.
- Neuroendocrine neoplasms (NENs) are rare neoplasms arising from cells of the diffuse endocrine system, mainly dispersed throughout the digestive system and respiratory tract. Most NENs grow slowly and symptoms may be related to tumour mass (non-functioning, NF-NENs) and/or to the hypersecretion of hormones (functioning, F-NENs). Histopathologically, most NENs are well-differentiated (WD) tumours grade 1 (G1, Ki67 ≤2%), grade 2 (G2, Ki67 3–20%) or grade 3 (G3, Ki67 >20%), whereas a small fraction are defined as poorly differentiated (PD) neuroendocrine small-cell or large-cell carcinoma (NEC) with a Ki67 >20% (5, 6).
- Patients with NENs and severe infections causing respiratory difficulties or gastrointestinal (GI) symptoms (e.g. diarrhoea, nausea and vomiting) are likely to present to hospital. Such ill patients may not be able to take their prescribed medication (e.g. analgesia, anti-diarrhoeals, proton pump inhibitors, oral chemotherapy drugs, targeted drugs), or the medication may not be absorbed, and these patients may require admission for treatment.
- Some patients presenting with Covid-19 infection may have an underlying undiagnosed NEN, and this possibility should be considered in those patients whose symptoms, for example, diarrhoea, or wheeze with shortness of breath, are not resolving, or whose symptoms were chronic.

## Management of patients with NENs and Covid-19 infection

- There are currently no drug-treatments or vaccines available for Covid-19, and therefore, managing patients with NENs alongside Covid-19 is likely to be an ongoing challenge for many months.

### General measures

- The general measures for treatment are the same as in any other patient with Covid-19 infection and should involve acute management of airway, breathing and circulation.

### Specific measures

**Figure 1:**
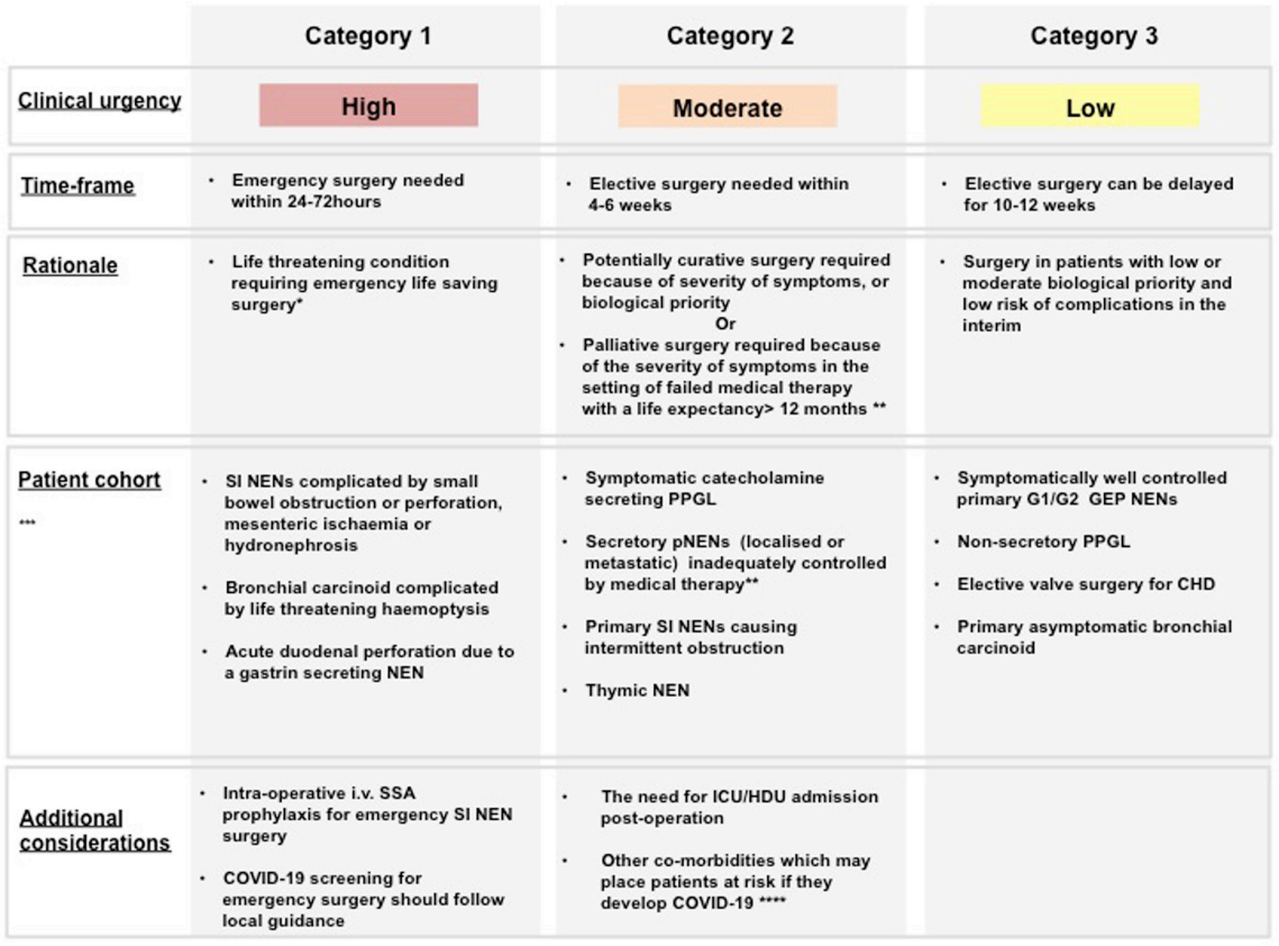
A suggested approach to surgical decision making during the Covid-19 pandemic for patients with NENs. *The patient's prognosis and life-expectancy from the underlying NEN must be considered in each case and discussed with the patient, family and surgical team. **Locally ablative therapies e.g. RFA, radio-embolisation or PRRT could be considered as an alternative to surgery in select patient's. ***This is not an exhaustive list but designed as a guide and all non-emergency surgical cases should be discussed at a specialist NEN MDT. ****Age over 60, pre-existing cardiovascular disease, pre-existing respiratory disease. CHD, carcinoid heart disease; PPGL, phaeochromocytoma/paraganglioma; pNEN, pancreatic neuroendocrine neoplasm; SI NEN, small intestine neuroendocrine neoplasm; PRRT, peptide receptor radionuclide therapy; SSA, somatostatin analogues; RFA, radiofrequency ablation; GEP, gastro-enteropancreatic

**Figure 2:**
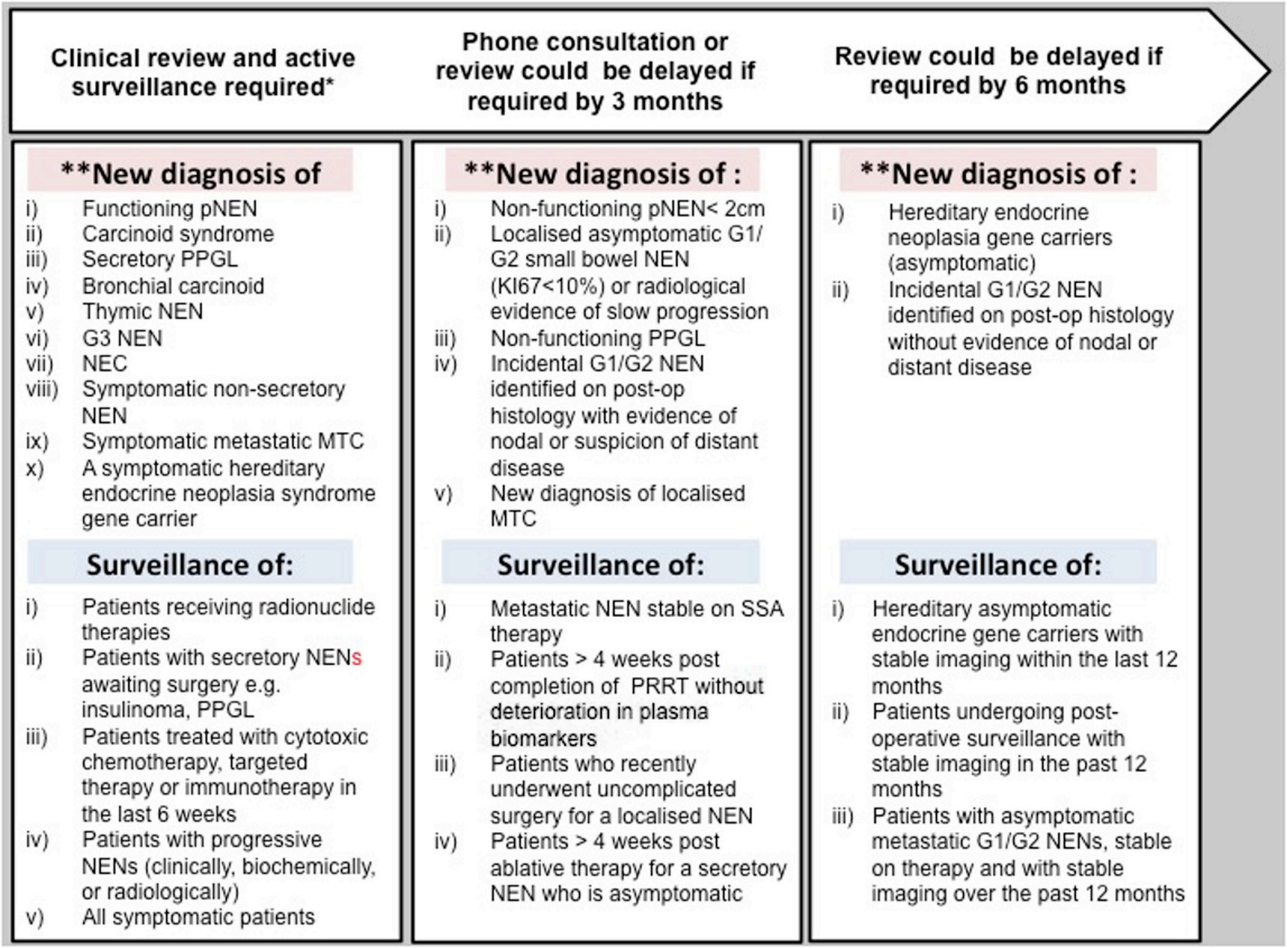
A suggested approach to the timing of clinical assessment during Covid-19 for new and follow-up patients with NENs. *The timelines and type of surveillance should follow local and ENETS guidelines where possible. **The diagnosis may be based on limited biochemical and radiological data and clinical judgement should be applied. PPGL, phaeochromocytoma/paraganglioma; pNEN, pancreatic neuroendocrine tumour; PRRT, peptide receptor radionuclide therapy; SSA, somatostatin analogues; NEC, neuroendocrine carcinoma; MTC, medullary thyroid carcinoma.

- Specific emergency treatments may need to be directed to the NEN (Table 1).
- Surgery (or endoscopic removal) is the only curative option for localised NENs (7). For those patients with compressive symptoms or acute complications, surgery may need to be considered during the Covid-19 pandemic (Fig. 1). For patients with localised disease who are asymptomatic, surgery could be reasonably delayed for 8–12 weeks.
- Patients with NENs who have had prior treatments (e.g. surgery, chemotherapy, everolimus, sunitinib, or radionuclide therapy) may have developed sequelae such as diabetes mellitus, or be on glucocorticoids, or be immuno-compromised, which will make them vulnerable to the severe complications of Covid-19 infection.
- NENs may arise in any organ, and those occurring in the respiratory tract (referred to as carcinoids), gastrointestinal (GI) tract and pancreas (referred to as GEP-NENs), thyroid, and adrenal (Table 1).
- NENs may occur with other tumours in patients with heritable endocrine tumour syndromes, such as multiple endocrine neoplasia (MEN) and von Hippel-Lindau disease (VHL). There are four major forms of MEN. In MEN1, pancreatic and lung (and rarely thymic) NENs occur with parathyroid tumours and anterior pituitary adenomas (8). In MEN2 (previously MEN2A), medullary thyroid carcinoma (MTC) occurs with phaeochromocytoma and parathyroid tumours, while in MEN3 (previously MEN2B) parathyroid tumours are rare, and the occurrence of MTC and phaeochromocytoma is found in association with a marfanoid habitus, mucosal neuromas, medullated corneal fibers, and intestinal autonomic ganglion dysfunction leading to megacolon (9). In the very rare MEN4, parathyroid adenomas, pituitary adenomas, and pancreatic NENs occur in association with gonadal, adrenal, renal and thyroid tumours. In VHL pancreatic NENs and PPGL occur in addition to haemangioblastomas and renal cell carcinoma.
- Based on our experience/knowledge gained from the consequences of other severe infections in patients with NENs, and on expert opinion, a brief overview of NENs for the non-specialist is given together with specific management recommendations for individual conditions (Figs 1 and 2) and service provision during the Covid -19 pandemic.

**Table 1:** Clinical manifestations, investigations and first-line treatments for NENs.

NEN	Clinical manifestations	Secreted hormones/compounds	Basic* tests (special tests)	Emergency treatments	Contraindicated drugs
Bronchial	As, CS, Ob (Cushing syndrome)	H, S, ACTH,GH	PlCgA, 5HIAA, ACTH, CT scan	SSA^g^/H1/H2 SX	
Thymic	As, CS, Ob (Cushing syndrome)	H, S, ACTH	PlCgA ACTH CT scan, SRI	SX, as often aggressive clinical behaviour needing prompt resection	
Pancreatic					
Gastrinoma	Haematemesis, epigastric pain, PU, diarrhea	Gastrin	Hb, U&E, LFTs, Ca^++^, gastrin, PlCgA endoscopy, CT scan, MRI, SRI	PPI H2 Transfusion SX	Aspirin NSAID
Insulinoma	Whipple's Triad	Insulin C-peptide	Glucose (overnight fast), (insulin, C-peptide), PlCgA, U&E, LFTs,FBC CT scan, MRI, EUS, SRI	Glucose (p.o. or i.v.) Diazoxide, SSA^g^, SX	
Glucagonoma	Wt loss, anaemia, stomatitis, rash-NME, venous thrombosis	Glucagon	Hb, U&E, LFTs, glucose, (glucagon), PlCgA, CT scan, MRI, EUS, SRI	SSA^g^ SX	
VIPoma	WDHA	VIP	Hb, U&E, LFTs, PlCgA CT scan, MRI, (VIP), EUS, SRI	SSA^g^ SX	
NF (PPoma)	As, Wt loss, Ob	PP or none	Hb, U&E, LFTs, (PP), PICgA, CT scan, MRI, EUS	SSA^g^ SX	
Small intestinal	As, CS, Ob, Wt loss	H, S, Cg A/B	Hb, U&E, LFTs, PlCgA, CT scan, MRI SRI, Urinary 5HIAA, Echo	SSA^g^/HI/H2/SX	Cyclizine, Adrenaline, Noradrenaline, D-tubocurarine, Atracurium, Morphine
Appendix	As, CS, Abdo pain	H, S, Cg A/B	Hb, U&E, LFTs, PlCgA, CT scan, MRI SRI	SX	
Colorectal	As, CS, Ob, Wt loss	H, S, Cg A/B	Hb, U&E, LFTs, PlCgA, CT scan, MRI SRI	SSA^g^/HI/H2/SX	
Phaeochromocytoma	↑ BP, headaches, palpitation, sweating, As	Adr, Nor	Hb, U&E, LFTs, Pl or Ur metanephrines, CT scan, MRI	Alpha blockade (p.o. or i.v.)/SX	Beta adrenoreceptor blockade (unopposed), Metoclopramide, Naloxone
Paraganglioma	↑ BP, headaches, sweating, palpitation, As, Lump(s)	Adr, Nor	Hb, U&E, LFTs, Pl or Ur metanephrines, CT scan, MRI, SRI	Alpha Blockade/SX	Beta adrenoreceptor blockade (unopposed)
MTC	Neck lump, As, Dysphagia, Diarrhoea, Flushing, (Cushing's syndrome)	Calcitonin (ACTH)	Calcitonin, CEA, Hb, U&E, LFTs, TFT's Ultrasound scan, CT scan, MRI	SX+/- systemic therapy	

^g^QT-prolonging drugs should be avoided in patients taking SSA therapy including medications which may be used as part of the treatment of Covid-19 e.g.; macrolides, azole anti-fungals, antimalarials, pentamidine, ciprofloxacin and moxifloxacin. If there are no alternatives to these medications, careful cardiac monitoring is required; *denotes basic tests that are likely to be available in most hospitals

↑BP, hypertension; 5HIAA, 5-hydroxyindoleacetic acid; Abdo, abdominal; ACTH, adrenocorticotropic hormone; Adr, adrenaline; alpha blockade, e.g., phenoxybenzamine or phentolamine; As, asymptomatic; Ca++, plasma calcium; CgA, chromogranin A; CS, carcinoid syndrome – facial flushing and odema, diarrhoea, abdominal pain, telangiectasia, carcinoid heart disease, wheezing, pellagra-like skin lesions; CT, computed tomography; EUS, endoscopic ultrasound; H, histamine; H2, histamine receptor 2 blocker (eg, ranitidine or cimetidine); Hb, haemoglobin; HI, histamine receptor 1 blocker (anti-histamine); LFTs, liver function tests; MRI, magnetic resonance imaging; MTC, medullary thyroid carcinoma; NEN, neuroendocrine neoplasm; NF, non-functioning; NME, necrolytic migratory erthyaemia; Nor, noradrenaline; NSAID, non-steroidal anti-inflammatory drugs; Ob, obstruction; Pl, plasma; PlCgA, plasma CgA; PPI, proton pump inhibitor (e.g. lansoprazole or omeprazole); PPoma, pancreatic polypeptide (PP) tumour; PU, peptic ulcer; S, serotonin; SRI, somatostatin receptor imaging; SSA, somatostatin analogue (eg, octreotide or lanreotide); SX, surgery; U&E, urea and electrolytes; Ur, urine; VIPoma, vasoactive intestinal peptide (VIP) tumour; WDHA, watery diarrhoea, hypokalaemia, achlorhydria; Whipple's triad, hypoglycaemic symptoms, blood glucose <2.2mmol/L, relief of symptoms following ingestion of glucose; Wt, weight.

## (i) Neuroendocrine neoplasms of the respiratory tract and thymus

### Bronchial carcinoids

- Bronchial carcinoid tumours can secrete serotonin, vasoactive substances and other hormones directly into the systemic circulation (Table 1) (8).
- Bronchial carcinoids may present with bronchial obstruction, cough, haemoptysis, weakness, nausea, weight loss, and neuralgia, or incidentally on chest imaging.
- Diffuse idiopathic pulmonary neuroendocrine cell hyperplasia (DIPNECH) can also cause acute respiratory symptoms and may be difficult to differentiate from Covid-19 infection on chest x-ray or conventional cross-sectional imaging. We advise prioritising Covid-19 testing for such patients. If viral testing is negative, a biopsy to provide a definitive diagnosis should be considered.

### Thymic neuroendocrine neoplasms

- Thymic NENs may cause compression of the trachea and mediastinal blood vessels and may secrete hormones.

## (ii) Gastrointestinal neuroendocrine neoplasms (GI NENs)

- GI NENs may be asymptomatic or they may present with obstructive symptoms (pain, nausea, vomiting), or with symptoms due to hormonal secretion.
- For major NEN surgery prophylactic perioperative treatment with i.v. octreotide, a somatostatin analogue (SSA), at a starting dose of 50–100 µg/h, is used to prevent carcinoid crisis (9) (see below), and drugs that stimulate the sympathetic nervous system or cause histamine release such as morphine and D-tubocurarine are avoided. Well-differentiated grade 3 GI NENs with a Ki-67 <55% should be treated as for grade 1 and 2 GI NENs unless there is evidence of rapid tumour growth, in which case they should be treated as for poorly differentiated NECs (10).
- Irrespective of Covid-19 risk, NECs should be treated urgently with appropriate chemotherapy (10, 11).
- NEC patients being treated with chemotherapy may be immunocompromised and therefore at high risk for Covid-19.
- If a NEC is discovered in a patient with severe Covid-19, the respiratory illness should be treated first, followed by early and appropriate chemotherapy following recovery.

### Small intestinal (SI) NENs

- SI NENs, which can produce serotonin and other vasoactive substances, may present with loco-regional disease or distant metastatic disease and are associated with estimated five-year survival rates of 65 and 35%, respectively (8).
- Loco-regional disease may present with abdominal pain due to mesenteric venous insufficiency, auto-infarction with tumour necrosis and haemorrhage, serotonin-induced retroperitoneal fibrosis, hydronephrosis or small bowel obstruction, which may require surgical intervention as an emergency.
- Metastatic and systemic disease may result in features of the carcinoid syndrome, which occurs in approximately 20% of SI NENs. Features of carcinoid syndrome include facial flushing, angioedema, diarrhoea, wheeze, ascites, pellagra and symptoms of right-sided heart failure caused by fibrosis of right-sided heart valves, so-called carcinoid heart disease (CHD). Some patients have lacrimation, rhinorrhoea, and episodic palpitations when they flush.
- The first-line therapy for patients with metastatic carcinoid is a long-acting SSA (e.g. octreotide LAR or lanreotide autogel) which has anti-proliferative and anti-secretory benefits in NENs (12, 13). Anti-diarrhoeal drugs (e.g. loperamide, codeine phosphate) and anti-histamines (blocking histamine 1 (H1) and 2 receptors (H2) for flushing may help some patients.
- At present there is no evidence to suggest that SSAs increase the risk of Covid-19 infection.
- SSAs can cause QT prolongation, and choice of anti-microbial and/or cardiac monitoring should be carefully considered for patients with Covid-19 taking SSAs (14) (Table 1).
- Treatment with telotristat ethyl may be considered in some centres for the management of refractory diarrohea in patients with carcinoid syndrome.
- A carcinoid crisis is a medical emergency caused by the sudden release of serotonin and other vasoactive substances into the systemic circulation and is characterised by intense flushing, bronchospasm, tachycardia, labile blood pressure (hypertension or profound hypotension) (15). A carcinoid crisis may look like an anaphylactic attack, but adrenaline must not be given as it will provoke, not help, carcinoid attacks (see below and Table 1) (15, 16).
- Common precipitants of a carcinoid crisis include: (i) intra-operative handling of the primary tumour; (ii) biopsy or ablative therapies of a tumour; (iii) anaesthetic induction; and (iv) specific medications such as cyclizine, long-acting vasopressors (e.g. noradrenaline) and drugs which stimulate histamine release (e.g D-tubocurarine) (Table 1).
- Treatment for a carcinoid crisis comprises an i.v. bolus of octreotide (25–500 µg) followed by an i.v. infusion of octreotide at a starting dose of 50–150 µg/h, together with i.v. fluids and appropriate cardiovascular measures to treat the likely intracardiac hypovolaemia and decreased pulmonary artery pressure (8, 7, 16).
- At present, there are no data regarding the risk of a carcinoid crisis in patients with carcinoid syndrome who develop acute viral infections, but patients with carcinoid heart disease may suffer significant decompensated heart failure.

### Large intestinal (LI) NENs

- LI NENs may present with obstructive symptoms (pain, nausea, constipation and diarrhoea), weight loss, or rectal bleeding, loco-regional disease similar to SI NENs, and rarely carcinoid syndrome (see above) (8).

## (iii) Pancreatic NENs

- PNENs may secrete endogenous hormones or be non-hormone secreting (non-functioning) (Table 1).
- Surgery is the treatment of choice for non-metastatic PNENs measuring 2 cm or greater in size, as it is often curative (7, 9).

### Insulinomas

- Hypoglycaemia in association with neuroglycopenia symptoms that are relieved by administration of glucose are the cardinal features (Whipple's triad) (Table 1) (9, 17).
- The combination of hypoglycaemic symptoms with documented hypoglycaemia (blood glucose <2.2 mmol/L) with hyperinsulinaemia (>30 pmol/L) and inappropriately increased circulating C-peptide (>300 pmol/L), in the absence of sulphonylurea or related drugs, in the plasma and urine is pathognomonic of insulinoma.
- Hydroxychloroquine as a treatment for Covid-19 can cause hypoglycaemia, which should be differentiated from hyperinsulinaemic hypoglycemia.
- Hypoglycaemia is an emergency. Initially in a conscious and cooperative patient, glucose 10–20 g (e.g. 2–4 teaspoons (or 3–6 lumps) of sugar, 150–200 mL of pure fruit juice, or 5–7 tablets of dextrose) can be given orally. In an unconscious patient i.v. administration of glucose is required (e.g. 50 mL of 20% glucose (dextrose) infusion over 10 min, into a large vein through a large-gauge needle since this concentration is irritant especially if extravasation occurs). Glucagon 1 mg i.m. may be used as an alternative.
- Frequent meals (or enteral feeding via nasogastric tube) with diazoxide can be started before proceeding more definitive treatment, for example, surgery.
- Somatostatin analogues (SSAs) can be effective, but need careful monitoring as they can worsen the hypoglycemia. Corticosteroids may benefit some patients.

### Gastrinomas

- Gastrinomas, which are most often located in the duodenum, but also occur in the pancreas, are associated with marked gastric acid production and severe peptic ulcers that are multiple and recurrent (Zollinger-Ellison syndrome). Some patients may also have diarrhoea (steatorrhoea) (9).
- Patients with severe haematemesis may need blood transfusions and i.v. fluid resuscitation, but for patients with Covid-19 and clinical or radiological evidence of pulmonary congestion, caution with i.v. fluid resuscitation is warranted.
- Medical treatment, which is directed to reducing gastric acid production, comprises high-dose proton pump inhibitors, which in severe cases may need to be combined with H2 blockers.

### Other PNENs

- The diagnosis and treatments of other PNENs is detailed in Table 1.

## (iv) Phaeochromocytoma and paragangliomas

- Phaeochromocytomas and paragangliomas (PPGL) may present with paroxysmal or sustained hypertension, and attacks of palpitations, tremor, perspiration, headache and anxiety (18).
- Patients with previously-treated PPGL should perform interval home monitoring of blood pressure and can be advised that their risk of Covid-19 is not increased. Regular review in asymptomatic patients can be delayed (Fig. 2).
- If admitted with Covid-19, no special requirements should be necessary, but plasma and urinary metanephrines may be grossly elevated during severe disease so should not necessarily be indicative of recurrent tumour.
- If a patient with Covid-19 is suspected of harbouring a PPGL and this has been biochemically proven, α-adrenoreceptor blockade should be instituted (oral with phenoxybenzamine or doxazosin, or i.v. phenoxybenzamine) with monitoring of blood pressure, and may be considered for β-adrenoreceptor blockade after adequate α-adrenoreceptor blockade (18).
- With adequate and appropriate sympathetic blockade, PPGL surgery could be postponed during an acute Covid-19 crisis (Fig. 1).

## (v) Medullary thyroid carcinoma (MTC)

- MTC may present as a palpable mass in the neck, which may be asymptomatic or associated with symptoms of pressure, dysphagia, diarrhoea or flushing.
- Diagnosis of MTC is based on histopathology (high calcitonin and or CEA may help but are not diagnostic)
- MEN2 patients with MTC may also have phaeochromocytoma, which must be excluded before undertaking any surgical intervention (19).
- Ectopic ACTH production by MTC may cause Cushing's syndrome.
- Metastatic MTC can be treated with limited surgical resection, external beam radiotherapy in certain situations, tyrosine kinase inhibitors (TKIs), SSAs or other agents.

## (vi) Specific recommendations for the management of NENs during Covid-19

### i. Multi-disciplinary meetings (MDT)

- Specialist NEN MDT meetings will be crucial during this period to ensure on-going governance and appropriate decision making. For each case, the risk of the proposed intervention, diagnostic procedure or treatment should be weighed up against the risk of hospital attendance and Covid-19.
- MDT meetings should have options for videoconference and virtual meetings.

### ii. Surgery for NENs

See Figure 1.

### iii. Clinical review for NEN patients

- Non face-to-face consultations including phone or video consultations for suitable patients is recommended (Fig. 2).
- Non-essential clinic consultations should be postponed (Fig. 2).
  Clinical review should be carried out with necessary safety precautions in place (Fig. 2).

### iv. Radiological surveillance for NEN patients

- Cross-sectional imaging should be considered for patients with suspected disease progression, based on clinical symptoms, biochemistry or anticipated tumour biology.
- Routine surveillance in asymptomatic patients, in whom suspicion of disease progression is low, could be delayed.
- Access to nuclear imaging may be limited by Covid-19 pandemic, and patients considered for peptide receptor radionuclide therapy (PRRT) with ^177^Lutetium-dotatate should be prioritised for somatostatin receptor imaging (SRI).

### v. Endoscopic procedures for NENs

- Endoscopy department closure or restriction may affect diagnosis of and interventions for NENs. SRI may be considered as an alternative to endoscopy-guided biopsy in certain cases. Individual centres should discuss alternative planning for emergency endoscopic procedures for patients with NENs during Covid-19.

### vi. Therapy for NENs

- Management of acute presentations due to secretory NENs is reviewed in Table 1.
- Patients with Covid-19 have an increased risk of venous thromboembolism (VTE) (20). We recommend that patients with NEN and Covid-19 receive VTE prophylaxis in accordance with local protocols unless there is a significant contraindication, in which case careful discussion with a haematology specialist is advised.
- Conservative fluid resuscitation is generally recommended for patients with Covid-19 (21), but we advise that the i.v. fluid requirements for patients with Covid-19 and secretory NENs (e.g patients with VIPoma and PPGL) is guided by careful clinical assessment, blood pressure, electrolytes and invasive monitoring when necessary.
- For patients on active therapy who develop Covid-19, we recommend that treatment is withheld during the illness with the exception of SSAs for symptomatic secretory NENs.
- Options for home administration of SSA should be explored with patients and family.
- SSA therapy should be commenced in the absence of confirmatory SRI when delayed during the Covid-19 pandemic.
- SSA therapy can be considered as a bridge to a postponed curative surgery in asymptomatic patients with localised well-differentiated and non-secretory NENs.
- High-dose SSA therapy may be considered as a potentially safer alternative to PRRT, targeted therapy, or chemotherapy in patients with progressive well-differentiated GEP NENs (22).
- PRRT with ^177^Lutetium- dotatate therapy and other radionuclide therapies (e.g. ^131^I-Metaiodobenzylguanidine (^131^I-MIBG)) should be discussed at specialist NEN MDTs. Radionuclide therapies should be strictly performed according to international protocols, and if cytopaenia develops treatment cycles may need to be delayed or postponed.
- Monitoring for patients receiving PRRT and other radionuclide therapies should continue according to local guidelines.
- Patients may develop treatment-related fevers, leucopenia and lymphopenia post-PRRT and ^131^I-MIBG therapy. The differential diagnosis of Covid-19 should be considered and testing prioritised to exclude this diagnosis.
- Cytotoxic chemotherapy should be discussed at specialist NEN MDTs and alternative lower risks options considered for each patient.
- Local ablative therapies should be considered as an alternative to surgery or systemic chemotherapies in suitable patients.
- External beam radiotherapy should only be considered in patients with symptomatic bone metastases not responding to conventional analgesia.

### vii. Discussing resuscitation status for NEN patients

- Discussions regarding resuscitation and escalation of care for patients with NENs and Covid-19 should, when possible, involve NEN specialists for advice on treatments, prognosis and end-of-life measures related to the underlying NEN.

### viii. Support and educational resources

- Advice and support for NEN patients and families is provided at: www.amend.org.uk, www.netpatientfoundation.org, and paradifference.org.

### ix. Collecting data on patients with NENs and Covid-19

- We recommend recruiting patients with a diagnosis of NEN and COVID-19 to a dedicated registry (e.g. https://endo-ern.eu/ese-and-endo-ern-launch-an-initiative-to-collect-data-on-patients-with-rare-endocrine-conditions-and-covid-19/) in order to better inform future management of this patient group during the Covid-19 pandemic.

## Disclaimer

Due to the emerging nature of the Covid-19 crisis this document is not based on extensive systematic review or meta-analysis, but on rapid expert consensus. This document should be considered as guidance only; it is not intended to determine an absolute standard of medical care. Healthcare staff needs to consider individual circumstances when devising the management plan for a specific patient.

## Declaration of interest

The authors declare that there is no conflict of interest that could be perceived as prejudicing the impartiality of this guidance.

## Funding

R T C is supported by a grant from GIST Support UK; and R V T is supported by a Wellcome Trust Investigator Award (grant number 106995/Z/15/Z), National Institute for Health Research (NIHR) Oxford Biomedical Research Centre Programme), and NIHR Senior Investigator Award (grant number NF-SI-0514–10091).
